# Analysis and Evaluation of Groundwater Pollution for Coastal Agricultural Waste Landfills

**DOI:** 10.3390/toxics14060518

**Published:** 2026-06-12

**Authors:** Deyue Sun, Panshu Ma, Tong Qi, Wei Chen, Qingjia Meng, Ruizhi Liu, Wenwen Li

**Affiliations:** Key Laboratory of Estuarine and Coastal Environment, Ministry of Ecology and Environment, Chinese Research Academy of Environmental Sciences, Beijing 100012, China; 17852032421@163.com (D.S.); 15662358067@163.com (P.M.); epde@foxmail.com (T.Q.); chen.wei@craes.org.cn (W.C.); mengqj@craes.org.cn (Q.M.); liuruizhi@craes.org.cn (R.L.)

**Keywords:** informal agricultural waste landfill, groundwater pollution, pollution analysis, human health risk assessment, principal component analysis

## Abstract

With the rapid urbanization of China, environmental risks posed by informal landfills, particularly those dominated by agricultural waste, are an urgent yet understudied concern. This study systematically monitored groundwater quality surrounding five typical informal agricultural waste landfills in a coastal Chinese city. Eight major pollutants were analyzed using pollution index evaluation, the health risk model and multivariate statistical methods. The results indicate one landfill as a high-priority concern, exhibiting a combined multi-index pollution pattern with an exceedance rate of 87.5%, where NO_3_^−^-N, F^−^, COD_Mn_, and total hardness are the dominant indicators. Another landfill showed high background levels and anthropogenic impacts. Total non-carcinogenic risk of all landfills is below 1 (negligible). Children face approximately twice the health risk of adults. The exposure risk through drinking water ingestion is three orders of magnitude higher than that from dermal contact, with NO_3_^−^-N contributing >90% of the total risk. Groundwater deterioration is primarily affected by geological conditions and seawater intrusion (52.31%), followed by agricultural activities and soil characteristics. Given these findings, priority attention should be directed to nitrogen-driven landfill and multi-index composite pollution landfill, with reinforced source tracing and control of NO_3_^−^-N, alongside long-term monitoring for regional groundwater protection.

## 1. Introduction

Informal landfills are defined as those constructed and operated in non-compliance with relevant national policies, laws, regulations, standards, and specifications [1]. Despite their illegality and well-documented environmental harm, such landfills remain widespread globally, primarily because they involve low upfront costs and simple operational procedures compared to regulated sanitary landfills [2]. Informal landfills represent a global environmental challenge, particularly in developing countries where open dumping remains the predominant waste disposal method due to low upfront costs and limited regulatory enforcement [3]. According to the International Solid Waste Association (ISWA), the 50 largest informal dumpsites worldwide affect the daily lives of 64 million people, with 37 of these sites located on or near coastlines, posing additional risks to marine and groundwater quality [4]. Their prevalence thus represents a major environmental management challenge rather than a viable solution. Relevant surveys indicate that 90% of the waste in China is disposed of through simple landfill methods [5]. This extensive disposal mode continuously releases pollutants even after landfill closure, exerting adverse impacts on surrounding soil and water resources and posing potential ecological and public health risks [6]. Currently, informal landfills are listed as priority control targets among the key sources of groundwater pollution in China [7,8]. In May 2018, the General Office of the State Council issued the Three-Year Action Plan for Rural Living Environment Improvement, which stipulated that the systematic remediation of informal waste dumping sites in rural areas should be largely completed by the end of 2020.

In terms of pollution mechanisms, leachate from informal landfills is a primary concern, as it contains high concentrations of persistent organic compounds and heavy metals that can deteriorate groundwater quality and degrade soil function after infiltrating into aquifers [9,10]. In addition to the direct impacts of leachate, groundwater is also influenced by various environmental factors, including the dissolution of hydrological strata, atmospheric precipitation, and interactions with surface water bodies [11]. For example, in cold regions, climate-induced thawing of deep-frozen soil has been shown to release dissolved nitrogen, leading to elevated nitrate levels independent of landfill sources [12]. In certain basins, agricultural production has been identified as a potential source of aluminum contamination [13]. Collectively, these findings underscore that groundwater pollution near informal landfills is often a complex mixture of leachate-derived contaminants and elevated background concentrations from natural or agricultural sources. Most existing studies have examined these factors in isolation or only in the context of domestic/industrial waste landfills. A critical knowledge gap remains regarding how these multiple pollution sources—landfill leachate, geological background, and agricultural activities—interact specifically in coastal areas where groundwater is naturally characterized by high salinity and hardness. According to the 2021 Bulletin of China’s Ecological Environment Status, the primary pollutants in groundwater are the three forms of nitrogen (ammonium, nitrates, nitrites), sulfates, and chlorides—all of which can originate from landfill leachate as well as agricultural or geological sources. Therefore, distinguishing between these sources is not straightforward and requires integrated analytical approaches, which the present study aims to provide by focusing on informal agricultural waste landfills in coastal regions.

Most existing studies have focused on informal landfills dominated by domestic, industrial or construction waste, while research on the impacts of informal landfills where agricultural waste constitutes the primary landfill material remains scarce. Agricultural waste is characterized by high moisture content, easy decomposition, and high nitrogen and phosphorus contents. Its leachate composition differs significantly from that of other waste types, potentially leading to unique pollutant release patterns and environmental behaviors. In addition, coastal areas present additional complexity due to seawater intrusion, which elevates the background salinity and hardness of groundwater. The interaction between landfill leachate and naturally saline groundwater may produce combined pollution effects that have not yet been systematically investigated. Therefore, it is urgent to conduct groundwater pollution analysis and risk assessment for informal agricultural waste landfills to address this knowledge gap in this field. In view of the characteristics of sodium-salt-type, high-hardness groundwater in coastal areas, this study selected eight key indicators—Na^+^, Cl^−^, SO_4_^2−^, NO_2_^−^-N, NO_3_^−^-N, F^−^, COD_Mn_, and total hardness—which exhibit relatively high detection concentrations in informal agricultural waste landfills, to perform pollution source apportionment and risk assessment.

Contaminants in groundwater are characterized by persistence, biological toxicity, and non-degradability. These substances can enter the human body through multiple exposure pathways, impairing organ functions and even inducing various diseases [14,15]. Therefore, conducting human health risk assessments for groundwater around landfills is particularly essential. The health risk assessment model developed by the United States Environmental Protection Agency (USEPA) is the most widely adopted method in research conducted in China. In this study, the single-factor evaluation method and the Nemerow comprehensive index method are applied to assess groundwater pollution levels. By incorporating regional geographical features and human physiological characteristics, several key parameters of the USEPA model are revised. The modified model is adopted to identify the health risks posed by groundwater contaminants at the selected informal agricultural waste landfills. Meanwhile, multivariate statistical methods, including Pearson correlation analysis and principal component analysis (PCA), are employed to clarify the potential sources of the target groundwater pollutants. This study aims to investigate groundwater quality and human health risks in coastal areas affected by informal agricultural waste landfills, and to systematically analyze the potential sources of groundwater contaminants. The research findings can provide scientific support for prevention and control of groundwater pollution associated with agricultural waste landfills.

## 2. Data Sources and Research Methods

### 2.1. Overview of the Study Area

The informal landfills in the study area are primarily composed of agricultural waste, along with a mixture of construction and domestic waste. All landfills were closed in 2021. Closure measures include covering the sites with high-density polyethylene (HDPE) membranes, installing gas extraction pipes and rainwater drainage ditches, and setting up leachate collection systems. Groundwater monitoring wells were installed, and water sampling was implemented in 2022. The well layout strictly followed the Regulation of Groundwater Monitoring Well Construction [16]. These landfills are predominantly surrounded by farmland and vegetable greenhouses. The local stratigraphy consists primarily of clayey sand, sandy clay, silt, and coarse sand and gravel. Shallow groundwater in this region is primarily recharged by atmospheric precipitation, lateral river runoff and farmland irrigation. Groundwater discharge occurs mainly through artificial extraction and natural evaporation, followed by slow lateral runoff from south to north.

### 2.2. Sample Collection and Testing

**Figure 1:**
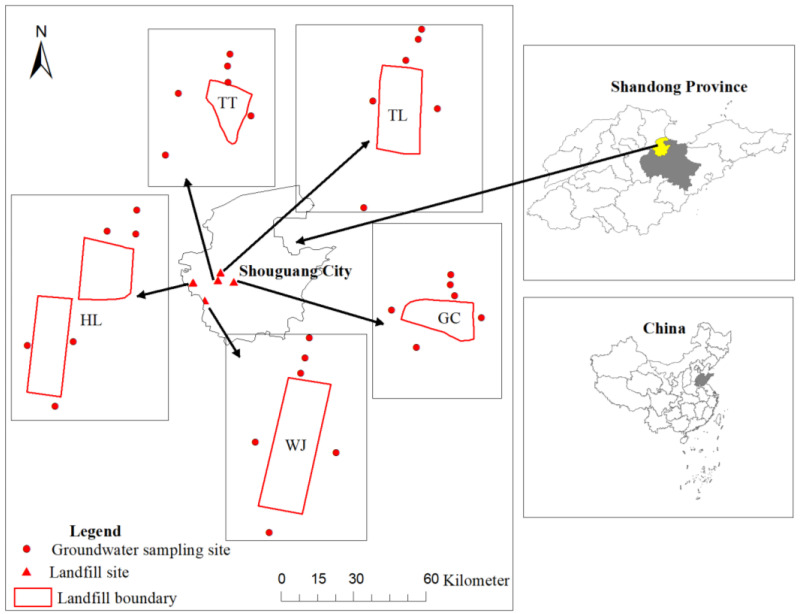
Location and distribution of the five informal agricultural waste landfills in the study area.

To analyze the impacts of informal agricultural waste landfills on surrounding groundwater, five typical informal (Figure 1) agricultural waste landfills are selected within the administrative region of the same coastal agricultural city in China. These landfills are distributed in different townships and spatially cover the main agricultural production zones of the study area. Those landfills are abbreviated as GC, HL, WJ, TT, and TL, respectively, based on the names of the townships in which they are located. For each landfill, five to six monitoring wells were installed in the surrounding area. Field sampling was conducted from May to July 2022 with groundwater depth below the surface ranged from 4.5 m to 29 m. Well purging was performed prior to sampling at each site, and three parallel samples were collected for every sampling point. All water samples were immediately stored in a vehicle-mounted refrigerator at 0~4 °C after collection and delivered to the laboratory for analysis within 24 h. All sample detection strictly followed the Technical Specifications for Groundwater Environmental Monitoring (HJ164-2020)  [17], and Table 1 lists analytical methods, instruments, and LODs for each parameter.

### 2.3. Quality Assessment Methods

#### 2.3.1. Method Accuracy and Applicability

The single-factor evaluation method and the Nemerow comprehensive index method are widely used for groundwater quality assessment due to their simplicity and effectiveness in identifying both individual pollutant exceedances and overall contamination levels. The single-factor method directly compares measured concentrations against standard limits, providing clear identification of specific pollutants of concern. The Nemerow method incorporates both the maximum and average pollution indices, thereby highlighting the most contaminated indicators while reflecting the overall pollution status. This combined approach has been demonstrated to be effective in landfill groundwater studies. In this study, all measurements were conducted following standard protocols (HJ164-2020) [17], and quality control measures (method blanks, duplicate samples, and certified reference materials) ensured data reliability. The coefficients of variation for replicate analyses were <5% for all parameters, indicating good analytical precision. The background reference values (Class III standards of GB/T 14848-2017) [18] are nationally recognized and appropriate for the study area where groundwater is primarily used for drinking and agricultural purposes.

#### 2.3.2. The Single-Factor Evaluation Method

The single-factor evaluation method reflects pollution level of each individual groundwater indicator. Table 2 listed evaluation criteria for the single-factor pollution index. The calculation method is as follows:

(1) $$\mathrm{Pi}\;=\frac{\mathrm{Ci}}{\mathrm{Bi}}$$

where *Pi* is the single-factor pollution index for indicator *i*; *Ci* is the measured concentration of indicator *i* in the water sample; and *Bi* is the corresponding permissible limit for indicator *i* according to the groundwater quality standard. Combined with the primary utilization purpose of groundwater in the study area, the Class III standard thresholds specified in the Groundwater Quality Standard (GB/T 14848-2017) [18] were adopted to evaluate the groundwater quality in this region. The Class III standard thresholds of Na^+^, Cl^−^, SO_4_^2−^, NO_2_^−^-N, NO_3_^−^-N, F^−^, COD_Mn_, and total hardness are 200, 250, 250, 1, 20, 1, 3 and 450 mg/L, respectively.

#### 2.3.3. The Nemerow Composite Index Evaluation Method

The Nemerow comprehensive index evaluation method is developed on the basis of the single-factor evaluation method. It incorporates both the maximum and the average values of the single-factor pollution indices, highlighting the indicators with severe contamination and thereby reflecting the comprehensive groundwater pollution status. Evaluation criteria for the comprehensive pollution index are listed in Table 1. The calculation method is defined as follows:

(2) $$P\;=\;\sqrt{\frac{P_{\mathrm{imax}}^{2}\;+\;P_{\mathrm{iave}}^{2}}{2}}$$

where *P* is the Nemerow comprehensive pollution index; *P_imax_* is the maximum single-factor pollution index among all indicators; and *P_iave_* is the average of all single-factor pollution indices.

### 2.4. The Health Risk Assessment Model

Based on the health risk assessment model proposed by USEPA [19,20,21], a chemical non-carcinogenic risk assessment was conducted for groundwater contaminants with potential adverse impacts on human health in the study area. Complete exposure assessment should include all relevant exposure pathways according to USEPA guidelines. According to surveys, groundwater pollutants mainly harm human bodies through two exposure pathways: dermal contact and drinking water ingestion [22,23]. The parameters in the model vary depending on regional and ethnic differences. In this study, we localized the USEPA health risk model parameters—including body weight, daily water intake rate, exposure frequency, and exposure duration—by referencing the Exposure Factor Handbook for Chinese Population and other relevant documents to reflect regional population characteristics [24]. This localized parameter revision approach is itself transferable: researchers in other countries or regions can apply the same health risk assessment framework by substituting their own locally validated exposure parameters. The methodological framework is universally applicable, thereby transforming our localized management conclusions into a global tool for groundwater health risk assessment at informal agricultural waste landfills. Non-carcinogenic exposure dose is calculated as follows:

(3) $$R\;=\;\frac{\mathrm{ADD}}{\mathrm{RfD}}\;\times \;10^{-6}$$

Here, R is the lifetime risk of equivalent death attributable to specific adverse health effects; ADD is the average daily exposure dose of non-carcinogenic pollutants (mg·kg^−1^·d^−1^); RfD is the reference dose for a chemical pollutant via a given exposure pathway (mg·kg^−1^·d^−1^); and 10^−6^ represents the corresponding assumed acceptable risk level. Exposure route via drinking water is calculated as follows:

(4) $$\mathrm{ADDd}\;=\;\frac{C\;\times \;\mathrm{IR}\;\times \;\mathrm{EF}\;\times \;\mathrm{ED}}{\mathrm{BW}\;\times \;\mathrm{AT}}$$

ADDd is the average daily exposure dose of non-carcinogenic pollutants through drinking water; IR is drinking water intake; C is the concentration of target contaminant in environmental media; EF is exposure frequency; ED is exposure duration; BW is body weight; AT is average exposure time. Exposure route via skin contact is calculated as follows:

(5) $$ADDs=\frac{C\times Kp\times ET\times CF\times SA\times EF\times ED}{BW\times AT}$$

ADDs is the average daily dose of exposure to non-carcinogenic pollutants via skin contact. Here, SA represents the skin contact surface area (cm^2^); Kp is the skin permeation constant of the compound (cm·h^−1^); ET denotes the exposure time (h·d^−1^); and CF is the volume conversion factor (1 L·1000 cm^−3^). Total non-carcinogenic risk value is calculated as follows:

(6) $$\mathrm{HQ}=\frac{\mathrm{ADDd}\;+\;\mathrm{ADDs}}{\mathrm{RfD}}$$

(7) $$\mathrm{HI}=\sum_{i=1}^{n}\mathrm{HQ}(i)$$

If HI ˂ 1, the non-carcinogenic health risk is regarded as absent or negligible; if HI ≥ 1, potential non-carcinogenic health risks are identified.

### 2.5. Statistical Analysis

The experimental data were organized using Excel 2025 software. ArcGIS 10.2 and GraphPad 10.1.2 were used to map the geographical location of the study area and the pollutant-related data of the landfills, respectively. Pearson correlation analysis was performed using SPSS 26.0 software on the groundwater quality indicators of the five landfills to measure the strength of linear relationships among variables. In addition, Principal component analysis with orthogonal rotation (Varimax) was performed on the eight groundwater pollutants using SPSS 26.0 software. Prior to PCA, the suitability of the data for factor analysis was verified using the Kaiser–Meyer–Olkin (KMO) test and Bartlett’s test of sphericity. Based on the Kaiser criterion, components with eigenvalues > 1 were retained. A forced three-factor solution was selected according to the scree plot from the initial run. Only factor loadings with absolute values > 0.6 on each component were extracted for source interpretation.

## 3. Results and Discussion

### 3.1. Descriptive Statistical Analysis

**Figure 2:**
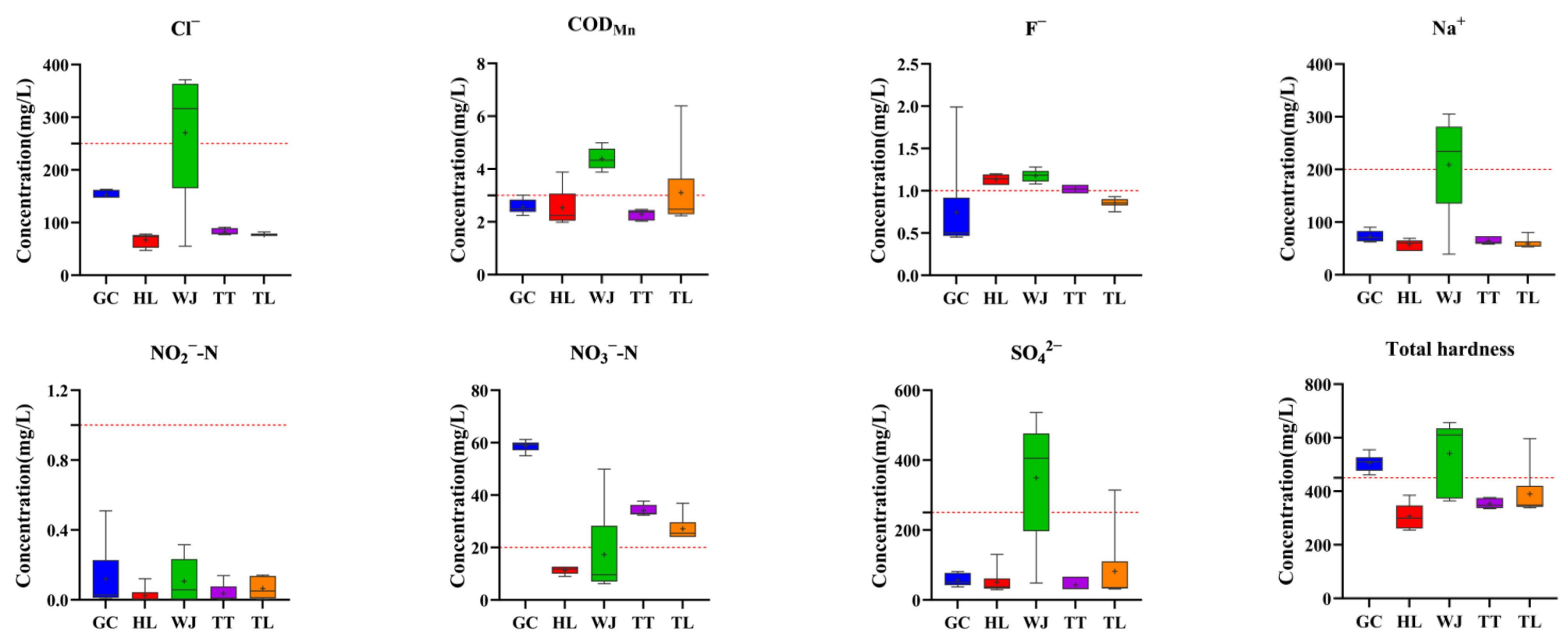
Groundwater pollution indicators were distributed in various landfills. The red dashed lines indicate the Class III standard limits for corresponding indicators as specified in GB/T 14848-2017 [18].

Descriptive statistical analysis was performed on the groundwater quality data collected from sampling sites of the five landfills in the study area to clarify the concentration characteristics of each chemical indicator. As shown in Figure 2, except for NO_2_^−^-N, the other seven indicators exceeded the corresponding standard limits to varying degrees, with over-standard rates ranging from 14.3% to 65.7%, indicating that regional groundwater had been adversely affected. Among all pollutants, NO_3_^−^-N exhibited the highest exceedance rate of 65.7%, followed by F^−^ with an exceedance rate of 51.4%. These two constituents were identified as the predominant contamination indicators in the study area.

NO_2_^−^-N exhibited a single-point anomaly distribution characteristic, with only the pollution monitoring well at the GC landfill showing a higher concentration than the control point. Moreover, this well had the highest concentration (0.51 mg·L^−1^) among all surveyed results. Other indicators in the well also showed higher concentrations than the control point. This anomaly was related to specific conditions at GC, suggesting that the well may have been influenced by the landfill. Based on the leachate data, the NO_2_^−^-N concentration in the leachate of the GC landfill ranged from 0.642 to 1.29 mg/L, which is higher than the value at the groundwater background control point. Moreover, the NO_2_^−^-N concentration in the groundwater at this anomalous sampling point is close to that in the leachate, further confirming the influence of localized leachate leakage. The reason might be the relatively short landfill time (less than one year), during which microbial activity was at its peak. Organic matter from above-ground waste is decomposed into small molecular compounds by microbial action and subsequently enters the groundwater, thereby increasing the concentrations of pollution indicators [25]. In the other landfills, groundwater NO_2_^−^-N concentrations were higher than leachate concentrations but remained within normal background ranges, and no similar anomalies were observed.

NO_3_^−^-N presented a characteristic of regionally high background values and possessed the highest overall exceedance rate of 66% among all detected indicators. Severe over-standard pollution of NO_3_^−^-N was observed in three landfills, namely GC, TT and TL, with a 100% exceedance rate. Combined with the values of NO_3_^−^-N at control sites of GC (59.4 mg·L^−1^), TT (32.7 mg·L^−1^) and TL (26.3 mg·L^−1^), it was found that the values were relatively high. In particular, the control points at GC and TL exhibited higher nitrate levels than those at the other monitoring and diffusion wells, indicating that the regional background concentration of NO_3_^−^-N in groundwater was inherently elevated rather than being derived solely from landfill leachate. By comparing the NO_3_^−^-N concentrations in leachate (mean leachate NO_3_^−^-N concentration: 6.92 mg/L at GC, 0.12 mg/L at WJ, and nearly undetectable in leachate from HL, TL, and TT), it was further confirmed that NO_3_^−^-N exceedances primarily originate from regional agricultural non-point source pollution rather than landfill leachate leakage. The excessive NO_3_^−^-N in groundwater is closely associated with geographical conditions and historical situation. Previous studies have proven that livestock breeding, domestic sewage discharge and excessive agricultural nitrogen input can aggravate groundwater nitrate contamination [26,27]. By contrast, all groundwater NO_3_^−^-N concentrations at the HL landfill all complied with the standard limits, which could be explained by the absence of residential areas and breeding farms in the surrounding region, resulting in fewer exogenous pollutant inputs.

COD_Mn_ exhibited a single-site dominance characteristic, closely related to human activity interference. COD_Mn_ primarily exceeded the standard at WJ with an exceedance rate of 100%. According to preliminary investigation results, the average COD_Mn_ value in the leachate at WJ was 3.78 mg·L^−1^, slightly lower than that in the groundwater, indicating the presence of other sources or accumulation effects in the groundwater besides direct leachate input. The overall higher coefficient of variation for various indicators at WJ compared to other landfills reflected a high degree of data dispersion, suggesting localized, intermittent external inputs rather than uniform background pollution. In contrast, leachate COD_Mn_ concentrations in the other landfills were higher than those in groundwater (particularly at the GC landfill, where leachate COD_Mn_ reached as high as 5.59 × 10^3^ mg/L). This pattern observed at WJ is highly consistent with the characteristics of anthropogenic disturbance, such as inadequate landfill closure or damage to the leachate collection system, indicating that the pollution at the WJ landfill might be related to human influence.

F^−^ exhibited a regionally prevalent elevation characteristic, with a total exceedance rate of 51.4%, second only to NO_3_^−^-N among landfills. The values of F^−^ in most landfills were relatively closely distributed with a small coefficient of variation, suggesting a relatively uniform and stable pollution source, possibly unrelated to human influence but related to other regional factors. Further comparison revealed that the F^−^ concentration in the groundwater at most of the five landfills was 2~4 times higher than that in the leachate, supporting the judgment that the primary source of F^−^ was geological rather than direct input from landfill leachate.

Except for the WJ landfill, the contents of Na^+^, Cl^−^ and SO_4_^2−^ presented consistent distributions without exceeding standard limits in other landfills. As critical hydrochemical indicators, these three ions can effectively reflect the regional hydrochemical characteristics of groundwater. This distribution feature indicates that the background values of saline ions in the study area are controlled by local geological and hydrological conditions, showing good spatial stability. By comparison, these indicators exhibited relatively high coefficients of variation and simultaneously exceeded the standard at several sampling points, while their concentrations in the leachate were generally undetectable, suggesting the possible presence of anthropogenic disturbance at WJ. From the perspective of leachate comparison at other landfills, the Cl^−^ concentration in the leachate of the GC landfill reached as high as 4.74 × 10^3^ mg/L, yet the groundwater Cl^−^ concentration remained stable between 147 and 165 mg/L, far below the leachate level, indicating no lateral migration of leachate. At the HL, TL, and TT landfills, Cl^−^ was undetectable in the leachate, and groundwater Cl^−^ concentrations were within the normal range. Therefore, Cl^−^ exceedances primarily indicate the influence of seawater intrusion rather than leachate leakage.

Multiple sampling sites with excessive total hardness were found in the GC and WJ landfills. The over-standard total hardness in the GC landfill coexisted with high NO_3_^−^-N concentrations, indicating a common pollution source mechanism for the two parameters. In the WJ landfill, the abnormal rise of total hardness was accompanied by the synchronous enrichment of Na^+^, Cl^−^ and SO_4_^2−^, which was closely related to anthropogenic disturbance. In contrast, although total hardness at the HL, TL, and TT landfills slightly exceeded the standard, groundwater total hardness concentrations were 7–10 times higher than leachate levels and were related to the high-salinity background caused by seawater intrusion rather than originating from landfill sources. The excessive total hardness was primarily attributed to the ion exchange between groundwater cations and soil Ca^2+^ and Mg^2+^, as well as the percolation of landfill leachate [28].

In summary, based on the groundwater contaminant exceedance data of the five landfills, the WJ landfill is associated with a high number of over-standard indicators, suggesting potential leachate leakage or intensive anthropogenic interference, and thus requires high-priority attention in subsequent management. The GC landfill exhibits a nitrogen-driven pollution pattern, with NO_3_^−^-N being the primary contributor to the comprehensive pollution index, necessitating strict source control of nitrogen pollution. In contrast, the HL, TT, and TL landfills demonstrate a regional background-dominated pollution pattern, where over-standards mainly result from high natural background levels. Therefore, their management strategies should focus on regional agricultural non-point source pollution control. Regarding the eight monitored indicators, NO_2_^−^-N, Na^+^, Cl^−^, and SO_4_^2−^ generally meet the groundwater quality standard requirements across most landfills. However, NO_3_^−^-N, F^−^, COD_Mn_, and total hardness show clustered exceedance in different landfills. Consequently, further source apportionment studies and targeted control measures are urgently required to address these issues.

### 3.2. Pollution Index Assessment and Health Risk Evaluation

**Figure 3:**
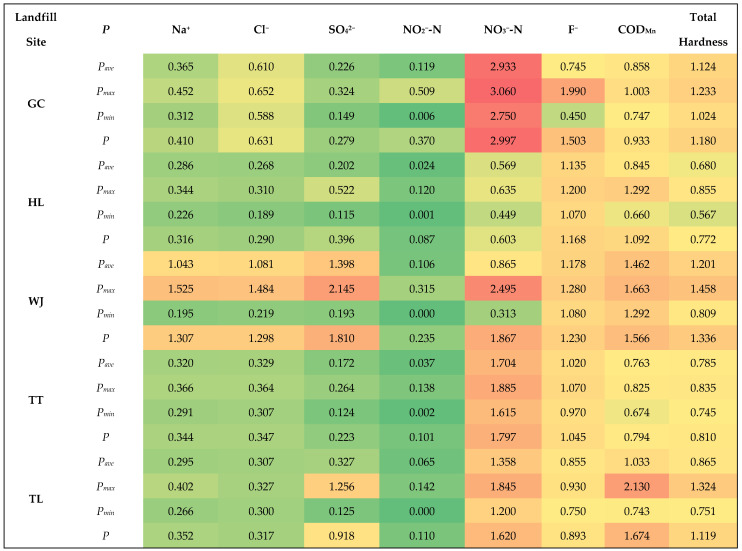
Color scale diagram of groundwater pollution index for landfill sites.

To further clarify the groundwater pollution characteristics of the landfills, the single-factor evaluation method and the Nemerow comprehensive index method were adopted to assess groundwater quality. Figure 3 listed the pollution index in five landfills, in which deeper shades represent higher numerical values. The single-factor pollution indices varied as follows: Na^+^ ranged from 0.23 to 1.53 with a mean of 0.55, Cl^−^ from 0.19 to 1.48 with a mean of 0.51, SO_4_^2−^ from 0.17 to 2.15 with a mean of 0.73, NO_3_^−^-N from 0.31 to 3.06 with a mean of 1.78, NO_2_^−^-N from 0 to 0.51 with a mean of 0.18, F^−^ from 0.45 to 1.99 with a mean of 1.17, COD_Mn_ from 0.66 to 2.13 with a mean of 1.21, and total hardness from 0.57 to 1.46 with a mean of 1.04. The mean values of single-factor pollution indices ranked in descending order as follows: NO_3_^−^-N > COD_Mn_ > F^−^ > total hardness > SO_4_^2−^ > Na^+^ > Cl^−^ > NO_2_^−^-N. These results indicated that NO_3_^−^-N served as the primary contributor to groundwater pollution in the study area, followed by COD_Mn_, F^−^ and total hardness, while Na^+^, Cl^−^, SO_4_^2−^ and NO_2_^−^-N presented relatively low pollution levels. Four indicators, including NO_3_^−^-N (except in HL), F^−^, COD_Mn_ and total hardness, maintained generally high concentrations, with slight to moderate pollution observed in all landfills. The comprehensive pollution indices of groundwater in the five landfills ranged from 0.93 to 2.24, with an average value of 1.5, suggesting an overall water quality ranging from clean to moderately polluted. The ranking of comprehensive pollution indices was GC (2.24) > WJ (1.62) > TT (1.36) > TL (1.34) > HL (0.93). The severe pollution in GC was mainly driven by the extremely high single-factor index of NO_3_^−^-N, which was closely associated with local geographical conditions and long-term agricultural activities. WJ had the largest proportion of indicators with slight to moderate pollution up to 87.5%, showing obvious characteristics of combined multi-index contamination. TT and TL showed moderate comprehensive pollution, primarily affected by NO_3_^−^-N and F^−^. By contrast, HL possessed the lowest comprehensive index and remained at a clean level, with only slight pollution of F^−^ and COD_Mn_. The above findings imply that differentiated control strategies should be adopted for different landfills: priority should be given to nitrogen pollution control at the nitrogen-driven landfill (GC), while collaborative control research on multiple indicators is required for the multi-index composite pollution landfill (WJ).

Human health risk assessment is a mathematical model based on groundwater quality standards and health-based reference values, which can quantitatively evaluate the potential hazards of groundwater contamination to human health. In accordance with the health risk parameters released by USEPA, three of the eight groundwater indicators in this study, namely nitrite nitrogen, nitrate nitrogen and fluoride, were identified to pose non-carcinogenic health risks to human beings. Their RfD values were 0.1 mg·kg^−1^·d^−1^ [29], 1.6 mg·kg^−1^·d^−1^ [29], and 0.04 mg·kg^−1^·d^−1^ [30], respectively. In this study, the non-carcinogenic health risks posed by the three contaminants were comprehensively evaluated via two primary exposure pathways: ingestion of drinking water and dermal contact.

**Figure 4:**
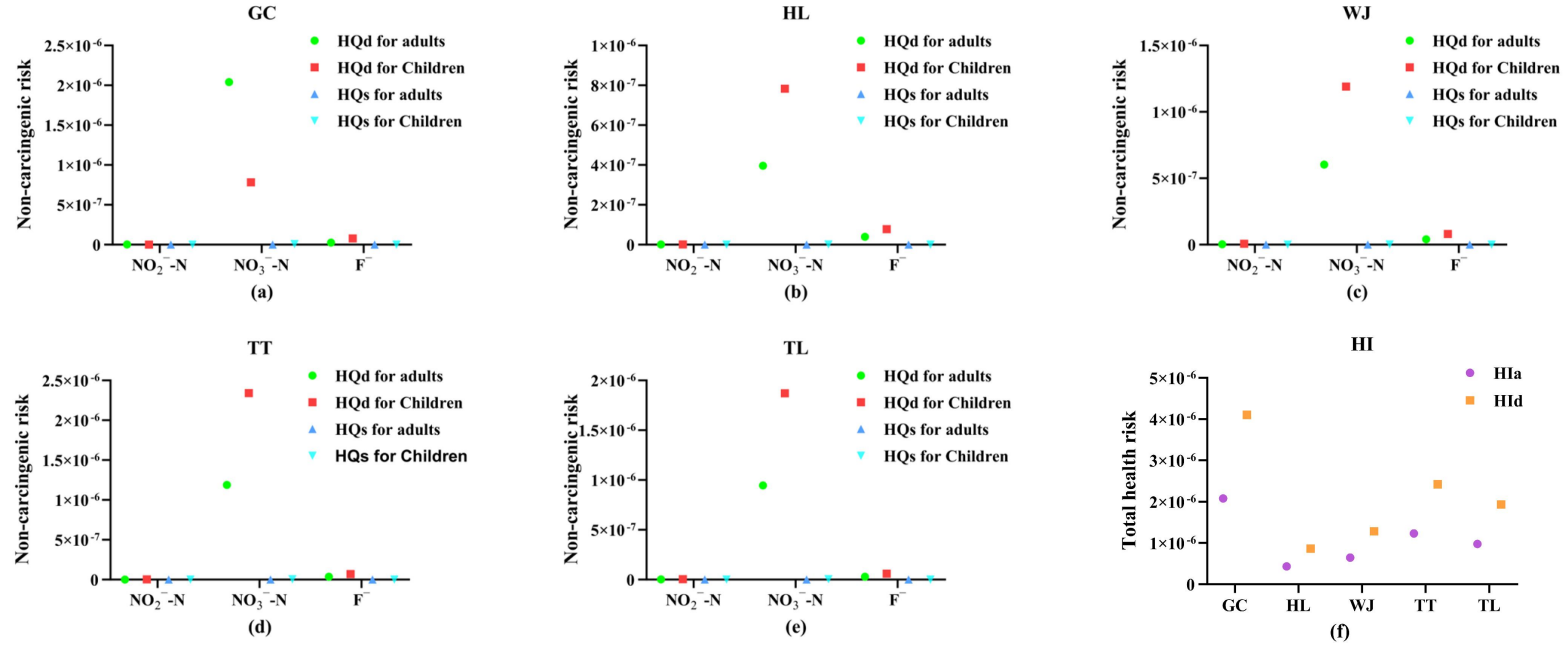
Annual average health risk due to pollution index in groundwater. (**a**–**e**) show the non-carcinogenic risk values of nitrite nitrogen, nitrate nitrogen, and fluoride at the five landfills (GC, HL, WJ, TT, and TL), respectively. (**f**) shows the total risk of the pollutants to adults and children across the five landfills. HQd refers to the exposure pathway via drinking water, while HQs refers to the exposure pathway via skin contact. HI represents the total non-carcinogenic annual health risk; HIa denotes the total non-carcinogenic annual health risk for adults; HId indicates the total non-carcinogenic annual health risk for children.

The health risks exhibit three distinct characteristics: drinking water as the dominant exposure pathway, NO_3_^−^-N contributing the most, and children being more sensitive. As shown in Figure 4, the health risks associated with drinking water (HQd for adults: 9.78 × 10^−7^–2.07 × 10^−6^; HQd for children: 8.62 × 10^−7^–4.09 × 10^−6^) at all landfill sites are significantly higher than those associated with skin contact (HQs for adults: 5.94 × 10^−10^–2.82 × 10^−9^, HQs for children: 1.59 × 10^−9^–7.56 × 10^−9^), with a difference of approximately three orders of magnitude, indicating that drinking water ingestion is the primary exposure pathway for human health risks in the study area. Under drinking water exposure, the trends in non-carcinogenic annual health risks for both adults and children are consistent, with NO_3_^−^-N (HQd for adults: 3.97 × 10^−7^–2.04 × 10^−6^; HQd for children: 7.83 × 10^−7^–4.04 × 10^−6^) > F^−^ (HQd for adults: 2.6 × 10^−8^–4.1 × 10^−8^; HQd for children: 5.12 × 10^−8^–8.1 × 10^−8^) > NO_2_^−^-N (HQd for adults: 8.37 × 10^−10^–4.16 × 10^−9^; HQd for children: 1.65 × 10^−9^–8.21 × 10^−9^). NO_3_^−^-N contributes more than 90% of the total risk, suggesting that among the three pollutants in the surveyed area, the health risks associated with NO_3_^−^-N warrant particular attention. The ranking of the total non-carcinogenic risks (Figure 4f) for the five landfill sites is GC (HI = 6.18 × 10^−6^) > TT (HI = 3.65 × 10^−6^) > TL (HI = 2.91 × 10^−6^) > WJ (HI = 1.93 × 10^−6^) > HL (HI = 1.3 × 10^−6^). The non-carcinogenic annual health risk caused by NO_3_^−^-N in the groundwater at GC is the highest. Under skin contact exposure, NO_3_^−^-N also poses the highest non-carcinogenic annual health risks for both adults and children. Therefore, among the five landfill sites (Figure 4a–e), the health impacts of the GC landfill site warrant attention. Analysis of the overall data reveals that for the five informal landfill sites, the risk values for non-carcinogenic pollutants in groundwater are generally higher for children than for adults, with children’s risks being approximately 2~3 times those of adults. This pattern aligns with the medical consensus that children have higher drinking water intake per unit of body weight than adults, possess immature physiological metabolic systems, and are more sensitive to pollutants [31].

The non-carcinogenic risks posed by groundwater pollutants at the five landfill sites are 2.08 × 10^−6^ (GC), 4.37 × 10^−7^ (HL), 6.48 × 10^−7^ (WJ), 1.23 × 10^−6^ (TT), and 9.79 × 10^−7^ (TL) for adults, and 4.1 × 10^−6^ (GC), 8.64 × 10^−7^ (HL), 1.28 × 10^−6^ (WJ), 2.42 × 10^−6^ (TT), and 1.93 × 10^−6^ (TL) for children. All values are well below the acceptable risk level recommended by USEPA, indicating that the current non-carcinogenic risks from groundwater pollutants at the landfill sites are negligible for both adults and children. Nevertheless, it is noteworthy that with the continuous accumulation of pollutants and sustained exposure to leachate from the landfill sites, the non-carcinogenic risks at the GC landfill site may increase, particularly the risks associated with NO_3_^−^-N, which warrant focused attention. The risk via the dermal contact pathway is negligible, and future monitoring efforts should focus on the drinking water ingestion pathway.

### 3.3. Analysis of Pollution Sources

Multivariate statistical methods have been widely applied to trace the sources of environmental pollution. In this study, correlation analysis and PCA were adopted to identify the potential sources of groundwater contaminants. Pearson correlation analysis was used as a preliminary step to explore pairwise relationships among groundwater contamination indicators (Table 3). While PCA can identify potential factors, correlation analysis provides complementary information on direct linear associations, which is particularly useful for confirming relationships between specific ion pairs (e.g., Na^+^ and Cl^−^) and for revealing negative correlations that PCA may fail to capture.

#### 3.3.1. Correlation Analysis

Pearson correlation analysis was performed on the pollution indicators of landfill groundwater in the study area. In general, indicators with significant correlation coefficients in groundwater are considered to share similar pollution sources and migration transformation processes [32]. As shown in Table 3, extremely significant positive correlations (*p* < 0.01) were observed between Na^+^ and Cl^−^, Na^+^ and SO_4_^2−^, and Cl^−^ and SO_4_^2−^, with correlation coefficients of 0.959, 0.949 and 0.882, respectively. These strong correlations indicate a high degree of common origin among the three ions, which are controlled by shared sources or similar migration and transformation conditions. Considering the hydrogeological setting of the coastal study area, these three ions likely arise primarily from the interplay of regional geological background and seawater intrusion. NO_3_^−^-N showed significant negative correlations with Na^+^, Cl^−^, and SO_4_^2−^, suggesting either source competition or geochemical constraints among these constituents.

This negative correlation reflects two potential mechanisms. On the one hand, agriculturally derived NO_3_^−^-N and geogenic salinity ions exhibit a spatially trade-off distribution pattern. On the other hand, high NO_3_^−^-N concentrations may inhibit the dissolution of fluoride-bearing minerals and disrupt cation exchange equilibrium during geochemical evolution. Additionally, nitrite nitrogen NO_2_^−^-N was significantly positively correlated with fluoride, suggesting partial source overlap or similar coexistence conditions. In combination with the previous conclusion that fluoride is predominantly of geogenic origin and the stable existence mechanism of nitrite nitrogen in alkaline soils, it is deduced that both indicators are jointly regulated by regional geological conditions and soil properties. COD_Mn_ exhibited a synergistic variation characteristic with total hardness, presenting an extremely significant positive correlation. Both parameters were also significantly positively correlated with Na^+^ and Cl^−^, and SO_4_^2−^, reflecting the combined natural and anthropogenic impacts on groundwater hydrochemical composition. This synergistic relationship indicates synchronous variations among organic pollution levels (COD_Mn_), hardness ions and salinity ions in groundwater. Leachate leakage may simultaneously introduce organic pollutants and soluble salts into the aquifer. Furthermore, organic acids produced during the degradation of organic matter can promote the dissolution of carbonate minerals, thereby increasing groundwater total hardness. Such coordinated variations among multiple indicators demonstrate that the hydrochemical characteristics of groundwater are jointly governed by natural processes (such as rock weathering and dissolution, seawater intrusion) and anthropogenic disturbances.

#### 3.3.2. Principal Component Analysis

PCA was further conducted to clarify the groundwater pollution sources of informal landfills in the study area [33]. As shown in Table 4, Principal component analysis with orthogonal rotation (Varimax) was performed on the eight indicators from the groundwater monitoring points of the investigated landfills (KMO = 0.501; Bartlett’s sphericity test, *p* < 0.001). Based on the eigenvalues from the initial scree plot, a forced three-factor solution was selected. The first principal component of the three-factor solution explained 52.307% of the variance, the second principal component explained an additional 19.304% of the variance (cumulative explained variance of 71.61%), and the third principal component explained an additional 17.481% of the variance (cumulative explained variance of 89.091%). Only factor loadings with high values (>0.5) on each component were extracted (Table 5). The results showed that Na^+^, Cl^−^, SO_4_^2−^, COD_M_ₙ, and total hardness had strong positive loadings on the first principal component; NO_3_^−^-N had a strong positive loading on the second principal component, while F^−^ exhibited a negative loading on the second principal component; NO_2_^−^-N and F^−^ exhibited positive loadings on the third principal component.

The contribution rate of PC1 was 52.307%, with high loading coefficients for Na^+^, Cl^−^, SO_4_^2−^, COD_Mn_, and total hardness, reaching 0.951, 0.921, 0.982, 0.752 and 0.822, respectively. COD_Mn_ can reflect the content of reducing substances in water bodies. Existing studies have demonstrated that groundwater COD_Mn_ is commonly affected by industrial wastewater discharge, agricultural activities and domestic sewage discharge [34]. Reductive organic matter contained in landfill leachate migrates into groundwater through soil leaching and a series of biochemical reactions. In addition, groundwater in the study area is weakly alkaline with a pH range of 7.4~7.8, a hydrochemical condition that facilitates the retention and stability of reducing substances, ultimately resulting in excessive COD_Mn_ concentrations. Additionally, Cl^−^ exhibits a certain degree of reducibility under acidic conditions, which can interfere with the determination of CODₘₙ. Therefore, CODₘₙ is, to some extent, influenced by the Cl^−^ concentration in water. Previous studies have shown that dissolution of subsurface salt deposits, lateral recharge from rivers, and industrial discharge may all contribute to anomalous concentrations of Na^+^, Cl^−^, SO_4_^2−^, and total hardness in regional groundwater [35]. In this study, the concentrations of the above indicators in the groundwater of the WJ landfill were relatively high with large variation coefficients, indicating that these pollutants were evidently affected by exogenous contamination to a certain extent. Except for the WJ landfill, the four indicators presented slight fluctuations in other landfills, suggesting stable and relatively single pollution sources. Such natural background contributions are mainly associated with soil leaching and water-rock interactions in deep groundwater [36]. Chen et al. [37] reported that the conventional ions in limestone aquifers (pH 8.1) mainly include Na^+^, Ca^2+^, Mg^2+^, Cl^−^, SO_4_^2−^ and HCO_3_^−^, among which SO_4_^2−^ dominates with the highest concentration, primarily controlled by gypsum dissolution. The study area is mainly covered by Tertiary gray limestone, with fluvo-aquic soil at a pH of 8.5. In recent decades, intense groundwater exploitation in coastal regions has disrupted the hydraulic balance between fresh water and seawater, triggering seawater intrusion. This process elevates the total hardness and ion contents of regional groundwater, particularly increasing the concentration of Cl^−^ with a fast migration rate [38,39]. According to the overall statistical results of the five landfills, the average concentration of Cl^−^ was higher than that of other ions including SO_4_^2−^. Consequently, the enrichment of Na^+^, Cl^−^, SO_4_^2−^ and total hardness in groundwater is predominantly attributed to geogenic processes, such as seawater intrusion and subsurface evaporite dissolution [28]. In summary, PC1 reflects the combined influence of regional geological conditions and seawater intrusion, overprinted by additional interference from landfill leachate—an effect particularly prominent at the WJ landfill.

The contribution rate of PC2 was 19.304%, which had a high loading for NO_3_^−^-N with a coefficient of 0.864. The contribution of F^−^ was the second highest, with a loading coefficient of −0.69. Generally, groundwater nitrate nitrogen is primarily derived from the discharge of domestic and livestock sewage as well as the application of nitrogen fertilizers in agricultural production [40]. Ammonia nitrogen is transformed into nitrate nitrogen through soil nitrification and eventually infiltrates into groundwater. In addition, geotechnical conditions also affect the migration of NO_3_^−^-N, loosely structured soil facilitates its downward leaching and infiltration [41]. The soil type in the study area is fluvo-aquic soil, which consists of humus horizon, redox horizon and parent material horizon. Nitrate nitrogen migrates downward alongside rock weathering and enters groundwater through ion exchange and adsorption in clay layers. Furthermore, high rainfall and intense evaporation in summer accelerate the migration of nitrate nitrogen in the soil, thereby exacerbating groundwater contamination [42]. Notably, the loading coefficient of NO_3_^−^-N at the GC landfill was markedly higher than that of other landfills, and its concentration at the control site also exceeded the standard threshold. This indicates that PC2 reflects not only the regional background of agricultural non-point source pollution but also the superimposed point-source contribution from landfill leachate. Under natural conditions, fluoride is widely distributed in soils and sediments. It readily binds with metal elements to form soluble compounds and migrates along with soil water [43,44]. Moreover, the dissolution of fluorine-rich rock formations during groundwater circulation serves as another critical source of fluoride in aquifers [45]. The local soil in the study area features high salinity and low calcium alkaline water, which provides favorable conditions for the dissolution of fluorine-bearing minerals [46]. Liu et al. [47] pointed out that fluoride contamination in groundwater of valley-type landfills originates from the combined effects of natural sources and landfill leachate. Combined with the results of this study, serious fluoride exceedance was observed in the groundwater of the HL and WJ landfills. The average fluoride concentrations in the leachate of the two landfills were 0.34 mg·L^−1^ and 0.30 mg·L^−1^, respectively, which were significantly lower than those in the corresponding groundwater. This demonstrates that fluoride enrichment is primarily controlled by intense natural leaching processes. Within the same study area, sporadic fluoride exceedance at individual monitoring points of the TT landfill may be closely related to groundwater runoff conditions. Slow groundwater flow facilitates fluoride accumulation, resulting in spot or patchy distribution of excessive fluoride [48,49]. The positive loading of NO_3_^−^-N and negative loading of F^−^ indicate source spatial differentiation: NO_3_^−^-N is linked to agricultural activities, whereas F^−^ originates from geogenic background, leading to a negative spatial correlation. In conclusion, this principal component is attributed to the combined effects of agricultural production activities and regional soil characteristics.

The contribution rate of PC3 was 17.481%, with high loading coefficients for NO_2_^−^-N of 0.937. The formation of nitrite nitrogen in groundwater is governed by soil nitrification and denitrification [50]. Under sufficient oxygen conditions, nitrifying bacteria drive nitrification, which oxidizes ammonia and nitrite into stable nitrate. Under anoxic conditions, denitrification occurs, through which denitrifying bacteria reduce nitrate to nitrite for energy metabolism. Soil in the study area is alkaline (pH 8.5), which inhibits nitrification by nitrifying bacteria and allows nitrite to remain stable within the soil matrix. Gradually, nitrite migrates into groundwater via soil leaching. In summary, PC3 is identified to be of geogenic origin, comprehensively controlled by regional lithology, soil properties and hydrogeological conditions.

## 4. Conclusions

This study systematically analyzed groundwater pollution characteristics and human health risks of five informal landfills in a coastal city of China. The results show that WJ landfill exhibited a multi-index composite pollution pattern, with the highest exceedance rate of 87.5% and large coefficients of variation, suggesting substantial leachate interference and anthropogenic disturbance. The GC landfill was characterized by nitrogen-driven pollution, with the highest comprehensive pollution index of 2.24, and NO_3_^−^-N contributed the dominant pollution load. By contrast, the HL, TT and TL landfills were of the regional background-dominated pollution type, where pollutant exceedance was mainly controlled by agricultural non-point source pollution and natural geological background. Overall, NO_3_^−^-N, F^−^, COD_Mn_ and total hardness were identified as the primary pollutants in the study area, and require targeted attention and priority control. For all landfills, the health risk via drinking water ingestion exceeded that from dermal contact. Currently, all non-carcinogenic risk values are below 1, indicating a negligible overall risk level. However, children are more sensitive than adults to contaminants, and the long-term cumulative risk cannot be ignored. Pollution source apportionment indicated that Na^+^, Cl^−^, SO_4_^2−^, COD_Mn_ and total hardness were mainly controlled by regional geological background and seawater intrusion, with a variance contribution rate of 52.31%. NO_3_^−^-N primarily originated from agricultural activities and soil properties, accounting for 19.30% of the total variance. F^−^ and NO_2_^−^-N were dominated by geogenic factors, with a contribution rate of 17.48%. Pearson correlation analysis further revealed distinct hydrogeochemical relationships: high homology among Na^+^, Cl^−^, and SO_4_^2−^; synergistic variation between COD_Mn_ and total hardness; and significant negative correlations between nitrate nitrogen and salinity ions.

Based on the identified pollution patterns, we propose differentiated management strategies: for the multi-index composite pollution landfill, long-term monitoring is recommended, with immediate engineering remediation to be implemented as warranted; for the nitrogen-driven landfill, priority should be given to nitrate source control and long-term monitoring; for the background-dominated landfills, regional agricultural non-point source management should be adopted. Additionally, alternative drinking water sources should be considered for households near high-risk sites to protect children, who face 2–3 times higher health risks than adults.

## Figures and Tables

**Table 1:** Analytical methods, instruments, and LOD for each parameter.

Parameter	Analytical Method	Instruments	LODs (mg/L)
Na^+^	Atomic absorption spectrometry	Atomic Absorption Spectrophotometer	0.01
Cl^−^	Ion chromatography	Ion Chromatograph	0.028
SO_4_^2−^	Ion chromatography	Ion Chromatograph	0.018
NO_2_^−^-N	Ion chromatography	Ion Chromatograph	0.064
NO_3_^−^-N	Ion chromatography	Ion Chromatograph	0.064
F^−^	Ion chromatography	Ion Chromatograph	0.006
COD_Mn_	Acidic potassium permanganate titration	Burette	0.4
Total hardness	EDTA titrimetric method	Burette	5
PH	Glass electrode method	pH Meter	/

**Table 2:** Evaluation criteria for the single-factor pollution index and the comprehensive pollution index.

*P*	Single-Factor Pollution Assessment	*Pi*	Comprehensive Pollution Assessment
*P* ≤ 0.7	Clean	*Pi* < 1	Clean
0.7 < *P* ≤ 1	Warning Level	1 < *Pi* ≤ 2	Slight Pollution
1 < *P* ≤ 2	Slight Pollution	2 < *Pi* ≤ 3	Moderate Pollution
2 < *P* ≤ 3	Moderate Pollution	*Pi >* 3	Severe Pollution
*P* > 3	Severe Pollution		

**Table 3:** Correlation matrix of groundwater pollution index concentrations in landfill.

	Na^+^	Cl^−^	SO_4_^2−^	NO_2_^−^-N	NO_3_^−^-N	F^−^	COD_Mn_	Total Hardness
Na^+^	1							
Cl^−^	0.959 **	1						
SO_4_^2−^	0.949 **	0.882 **	1					
NO_2_-N	0.022	0.121	0.050	1				
NO_3_-N	−0.390 *	−0.166	−0.392 *	0.244	1			
F^−^	0.234	0.119	0.250	0.475 **	−0.449 *	1		
COD_Mn_	0.568 **	0.485 **	0.763 **	0.155	−0.213	0.266	1	
Total hardness	0.721 **	0.827 **	0.768 **	0.292	0.231	−0.021	0.584 **	1

Note: ** Significant correlation at the 0.01 level (two-tailed). * Significant correlation at the 0.05 level (two-tailed).

**Table 4:** Variance accumulation of principal components of groundwater pollution index concentrations in landfill.

Component	Initial Eigenvalues			Extraction Sums of Squared Loadings			Rotation Sums of Squared Loadings		
	Total	% of Variance	Cumulative %	Total	% of Variance	Cumulative %	Total	% of Variance	Cumulative %
1	4.185	52.307	52.307	4.185	52.307	52.307	4.034	50.420	50.420
2	1.544	19.304	71.610	1.544	19.304	71.610	1.621	20.256	70.677
3	1.398	17.481	89.091	1.398	17.481	89.091	1.473	18.415	89.091
4	0.580	7.249	96.341						
5	0.209	2.609	98.950						
6	0.066	0.822	99.773						
7	0.016	0.203	99.976						
8	0.002	0.024	100.000						

**Table 5:** Principal component analysis of groundwater pollution index concentrations in landfill.

Pollution Index	1	2	3
Na^+^	0.951		
Cl^−^	0.921		
SO_4_^2−^	0.982		
NO_2_^−^-N			0.937
NO_3_^−^-N		0.864	
F^−^		−0.690	
COD_Mn_	0.752		
Total Hardness	0.822		

## Data Availability

The raw data supporting the conclusions of this article will be made available by the authors on request.
